# Transplanted hair follicle mesenchymal stem cells alleviated small intestinal ischemia–reperfusion injury *via* intrinsic and paracrine mechanisms in a rat model

**DOI:** 10.3389/fcell.2022.1016597

**Published:** 2022-10-05

**Authors:** Yang Gao, Haoyuan Chen, Xueyu Cang, Hongliang Chen, Yuzhu Di, Jihan Qi, Huimin Cai, Kunpeng Luo, Shizhu Jin

**Affiliations:** Department of Gastroenterology and Hepatology, The Second Affiliated Hospital, Harbin Medical University, Harbin, China

**Keywords:** small intestinal ischamia-reperfusion injury, hair follicle mesenchymal stem cells, homing, differentiation, paracrine

## Abstract

**Background:** Small intestinal ischemia-reperfusion (IR) injury is a common intestinal disease with high morbidity and mortality. Mesenchymal stem cells (MSCs) have been increasingly used in various intestinal diseases. This study aimed to evaluate the therapeutic effect of hair follicle MSCs (HFMSCs) on small intestinal IR injury.

**Methods:** We divided Sprague–Dawley rats into three groups: the sham group, IR group and IR + HFMSCs group. A small intestinal IR injury rat model was established by clamping of the superior mesenteric artery (SMA) for 30 min and reperfusion for 2 h. HFMSCs were cultured *in vitro* and injected into the rats through the tail vein. Seven days after treatment, the intrinsic homing and differentiation characteristics of the HFMSCs were observed by immunofluorescence and immunohistochemical staining, and the paracrine mechanism of HFMSCs was assessed by Western blotting and enzyme-linked immunosorbent assay (ELISA).

**Results:** A small intestinal IR injury model was successfully established. HFMSCs could home to damaged sites, express proliferating cell nuclear antigen (PCNA) and intestinal stem cell (ISC) markers, and promote small intestinal ISC marker expression. The expression levels of angiopoietin-1 (ANG1), vascular endothelial growth factor (VEGF) and insulin growth factor-1 (IGF1) in the IR + HFMSCs group were higher than those in the IR group. HFMSCs could prevent IR-induced apoptosis by increasing B-cell lymphoma-2 (Bcl-2) expression and decreasing Bcl-2 homologous antagonist/killer (Bax) expression. Oxidative stress level detection showed that the malondialdehyde (MDA) content was decreased, while the superoxide dismutase (SOD) content was increased in the IR + HFMSCs group compared to the IR group. An elevated diamine oxidase (DAO) level reflected the potential protective effect of HFMSCs on the intestinal mucosal barrier.

**Conclusion:** HFMSCs are beneficial to alleviate small intestinal IR injury through intrinsic homing to the small intestine and by differentiating into ISCs, via a paracrine mechanism to promote angiogenesis, reduce apoptosis, regulate the oxidative stress response, and protect intestinal mucosal function potentially. Therefore, this study suggests that HFMSCs serve as a new option for the treatment of small intestinal IR injury.

## 1 Introduction

Ischemia-reperfusion (IR) injury refers to the situation that the degree of tissue injury increases rapidly after the ischemic tissue and/or cells recover blood flow (reperfusion). Intestinal IR is a common injury in intestinal surgery, post-traumatic shock resuscitation, cardiopulmonary insufficiency, acute and chronic mesenteric vascular ischemia and mesenteric venous thrombosis (Grootjans et al., 2016; Mi et al., 2021). Small intestinal IR injury may eventually result in systemic inflammatory response syndrome (SIRS) and multiple organ dysfunction syndrome (MODS), both of which can be life threatening (Li et al., 2020; Li et al., 2021; Zheng et al., 2022). Clinically, the treatment of intestinal IR injury consists of supporting symptomatic treatment, maintaining haemodynamic stability, maintaining sufficient oxygen saturation and correcting electrolyte balance disorder (Gutiérrez-Sánchez et al., 2021). Drugs that target the mechanism of IR are still under development and have not been effectively applied in the clinic (Gonzalez et al., 2015; de Holanda et al., 2021). As a consequence, it is crucial to find new and effective methods to reduce intestinal IR injury.

Numerous researchers have used mesenchymal stem cells (MSCs) as potential therapeutic agents in intestinal diseases, and the use of MSCs as therapeutic agents is a key research topic in the regenerative medicine field. For example, bone marrow MSCs (BMMSCs) reduce mortality after intestinal IR injury (Markel et al., 2015). MSCs derived from amniotic fluid and fat have also been proven to repair intestinal IR injury (Watkins et al., 2013; Liu et al., 2020). Hair follicle MSCs (HFMSCs) are members of the stem cell family derived from hair follicles. They possess the characteristics of MSCs and can differentiate into osteocytes, adipocytes, smooth muscle cells, haemocytes and so on (Wang et al., 2020). Compared with MSCs from other sources, HFMSCs are easier to obtain from patients; additionally, the acquisition of HFMSCs causes little damage, and the cost of HFMSC acquisition is low. Moreover, HFMSCs exhibit strong differentiation ability and the potent ability to expand *in vitro* and can be directly applied to patients (Li et al., 2015; Savkovic et al., 2021). Previous studies on the treatment of other diseases with HFMSCs have shown that HFMSCs can home to the damaged tissue and reduce the damage caused by pancreatitis (Sun et al., 2021). Genetically modified HFMSCs were found to differentiate and exert endocrine signalling in improving ulcerative colitis (Zhou et al., 2021). HFMSCs also have the potential to improve ischaemic stroke (Zhang et al., 2020). However, to our knowledge, HFMSCs have not been applied to small intestinal IR injury. In view of the advantages of HFMSCs, we hypothesized that they would be a good cell type for small intestinal IR injury therapy.

In this study, we explored the therapeutic effects of transplanted HFMSCs on small intestinal IR injury to carry out a preliminary exploration to promote further research on HFMSCs in the treatment of small intestinal IR injury in the future.

## 2 Materials and methods

### 2.1 Experimental animals

Healthy male Sprague-Dawley rats, weighing 180–200 g, were used to establish the model used in this experiment. The experimental animals used to extract HFMSCs were male rats aged 8–10 days. We purchased experimental rats from the Animal Experimental Center of the Second Affiliated Hospital of Harbin Medical University, China. The animals were kept in a 12/12 h light-dark cycle chamber 22 ± 2°C. We fed rats with standard laboratory rodent diets and provided them with free access to water. However, experimental rats were fasted for 12 h before surgery. The experimental design and methods complied with ethical guidelines and standards, and were approved by the Ethics Committee of Harbin Medical University (approval number: SYDW 2019-226).

### 2.2 Experimental design

**FIGURE 1:**
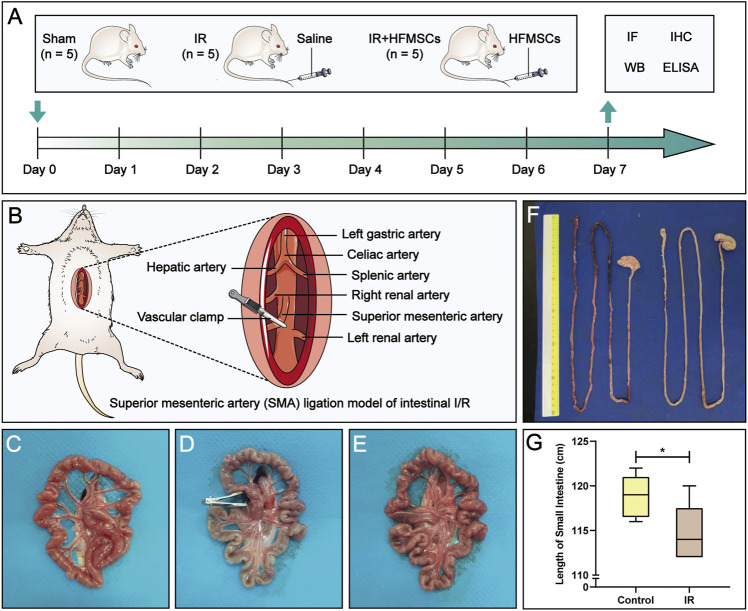
Experimental design and macroscopic evaluation of a small intestinal IR injury model. **(A)** The experimental animals were divided into three groups: the sham group (*n* = 5), IR group (*n* = 5) and IR + HFMSCs group (*n* = 5). Immunofluorescence analysis, immunohistochemistry, Western blotting, and ELISA were performed on day 7 to evaluate the therapeutic effect of HFMSCs. **(B)** The superior mesenteric artery (SMA) was clamped and opened to establish an intestinal IR injury model. **(C–E)** The morphology of the small intestine before **(C)**, during **(D)**, and after **(E)** SMA clamping is shown. **(F)** Small intestinal length was compared between the control group (*n* = 5) and the IR group (*n* = 5). **(G)** A statistical comparison of small intestinal length was performed. Data are presented as the mean ± SD (^*^ *p* < 0.05).

We divided the experimental animals into three groups. They were the sham group (*n* = 5), IR group (*n* = 5) and IR + HFMSCs group (*n* = 5) (Figure 1A). We established small intestinal IR injury model by clamping and opening the superior mesenteric artery (SMA). In the sham group, only laparotomy was performed, the SMA was isolated, and the abdomen was closed two and a half hours later. In the IR group, the SMA was clamped for 30 min, and reperfusion was applied for 2 h, followed by the injection of 1 ml of saline into the tail vein. Rats in the IR + HFMSCs group were subjected to intestinal IR *via* operation, and the same volume of a suspension containing 1×10^6^ ml^−1^ PKH67-labelled HFMSCs was injected into the tail vein. Previous experiments have proven that the proportion of MSCs that home to the small intestine after IR is higher on the 7th day than before or after the 7th day (Jiang et al., 2011; Jiang et al., 2013). After 7 days, immunofluorescence, immunohistochemistry, Western blotting and enzyme-linked immunosorbent assay (ELISA) were performed to evaluate the therapeutic effect of HFMSCs.

### 2.3 Small intestinal IR injury model establishment

The classic model of small intestinal IR injury was achieved by clamping the SMA (Gonzalez et al., 2015) (Figure 1B). After comparing and evaluating the IR modelling times used in previous studies, 30 min of SMA clamping and 2 h of reperfusion were used in this study (Jia et al., 2020; Hu et al., 2022). The rats were anaesthetized by intraperitoneal injection of a compound anaesthetic composed of 4.25% chloral hydrate and 0.886% sodium pentobarbital (0.3 ml/100 g). After approximately 5–10 min, the rats lost consciousness, and were then fixed in the supine position on an operating plate. After the skin on the abdominal surgical area had been prepared and disinfected, a longitudinal incision of approximately 5 cm was made on the midline of the abdomen. Sterile forceps were used to separate the SMA, and an atraumatic vascular clamp was used to clamp the SMA root for 30 min. During this period, sterile gauze soaked with saline was used to cover the surgical incision on the experimental animals to keep it moist. The vascular clamp was opened 30 min later, and the incision was sutured, and the abdominal cavity was closed 2 h later. During modelling, a small amount of warm saline was intermittently injected into the abdominal cavity intermittently to avoid transient hypovolemic reaction, and thermostatic pads were placed under the rats to maintain the body temperature at 37 ± 5°C.

### 2.4 Small intestinal IR injury model evaluation

We compared the control group (*n* = 5) and IR group (*n* = 5) based on the following three indicators to judge the success of model establishment. First, the lengths of the small intestine in the control and IR groups were compared. Rats were sacrificed after modelling, and the length of the small intestine was measured. Second, haematoxylin-eosin (HE) staining of the intestinal tract over a length of approximately 5 cm at 15 cm above the distal ileum was performed. More specifically, HE staining was carried out as follows: small intestines were dried with filter paper, fixed with 4% paraformaldehyde and then paraffin sectioned for approximately 4 μm thick. The sections were placed in a mixture of haematoxylin and eosin for even staining, and then placed in a constant temperature oven at approximately 60°C. After that the sections were placed into warm (40°C) water for washing, blow dried, and treated with xylene to complete dewaxing and clearing. Then the sections were sealed with neutral gum. Five pathological sections from each rat were selected for microscopic observation and photography with an optical microscope (Nikon Corp. Tokyo, Japan), and the microscopic fields of the sections were evaluated by calculating Chiu’s score (Chiu et al., 1970), a score specifically used to evaluate the degree of damage to the small intestinal mucosa. We invited two pathologists to score the sections in a double-blind manner. Third, we judged the height and area of small intestinal villi under one field of vision in each section. We selected five villi from the visual field and calculated their average villi height and area with Image-Pro Plus 6.0 and WPS Excel software.

### 2.5 HFMSC isolation and culture

At 8–10 days after the rats were sacrificed, the skin tissue at the whiskers was cut off and then cut into smaller strips along the area of hair follicles, with a row of hair follicles visible in each skin tissue sample, which was convenient for subsequent tissue digestion and HFMSC extraction. After repeated washing with phosphate-buffered saline (PBS), the skin tissue strips were placed in 0.1% type I collagenase (Sigma Aldrich, St. Louis, MO, United States) at 4°C in the dark for digestion for 15 h to more easily completely detach the hair follicles from the tissue. After sufficient digestion, the tissue was placed under a stereomicroscope (SZX7, Olympus, Japan), and the hair follicles were separated by microscopic tweezers and cultured in 24-well plates at 37°C and 5% CO2. The HFMSC medium consisted of DMEM/F12 (Sigma Aldrich, St. Louis, MO, United States), foetal bovine serum (ScienCell, San Diego, California, United States) and penicillin/streptomycin (Beyotime, Shanghai, China) at a ratio of 90:10:1. We observed the growth of adherent cells every day, and the culture medium was changed once every 1–2 days. The nonadherent cells were also discarded along with the discarded culture medium. When the primary cells covered 80% of the pore plate (approximately 2 weeks), the cells were passaged, and the second-passage cells were stained and injected. A flow chart is shown in Figure 3A. All the above protocols were carried out in a sterile environment.

### 2.6 HFMSC identification and staining

We evaluated the differentiation ability of HFMSCs based on whether they could be induced to undergo osteogenic and adipogenic differentiation. HFMSCs were cultured in osteogenic and lipogenic differentiation media for 14 days. Then Oil Red O (Sigma Aldrich, St. Louis, MO, United States) and Alizarin Red S (Sigma Aldrich, St. Louis, MO, United States) were used for HFMSC staining.

Flow cytometry was used by our research group to determine whether the HFMSCs extracted by the above methods could express the MSC markers CD29 and CD90; the HFMSCs expressed hardly any CD31 and CD45 (Sun et al., 2021). Therefore, HFMSCs were successfully extracted with this method.

Second-passage HFMSCs were stained with the green fluorescent cell membrane dye PKH67 (Sigma Aldrich, St. Louis, MO, United States). HFMSC nuclei were stained with DAPI (Beyotime, Shanghai, China). Cell staining was completed according to the instructions for the reagent. The stained HFMSCs were viewed using fluorescence microscopy (BX51, Olympus, Japan).

### 2.7 Immunofluorescence

After the experimental animals had been sacrificed, the small intestinal tissues were taken and washed with saline, and the surface was then dried with filter paper to remove moisture. The tissue blocks were cut into approximately 1 cm^3^ pieces and placed in an embedding box with optimal cutting temperature (OCT) compound wrapping the tissue. After that, the embedding box was slowly placed in liquid nitrogen to freeze, and placed in a −80°C freezer until use. A frozen slicer was used to cut the frozen tissue into 5-μm slices. 0.1% Triton X-100 was dropped on the sections to permeabilize cell. Then, the sections were sealed with goat serum (ABS933, Absin, Shanghai, China) at 37°C for 30 min, and primary antibodies were added and incubated at 4°C overnight. The primary antibodies used were anti-PCNA antibody (1:400, 13110, Cell Signaling Technology, Boston, United States), anti-Bmi1 antibody (1:100, ab126783, Abcam, Cambridge, United Kingdom), and anti-SOX9 antibody (5 μg/ml, ab185966, Abcam, Cambridge, United Kingdom). On the second day, goat anti-rabbit secondary antibody (SA00013-4, Proteintech, Wuhan, China) was added to the sections and incubated at 37°C for 1 h in the dark. Then, DAPI was added to stain the nuclei for 5 min. After that, images were taken by laser scanning confocal microscopy (Carl Zeiss Microscopy GmbH, Jena, Germany). The confocal cross-section fluorescence intensity was analysed with Origin 2017 and ImageJ software.

### 2.8 Immunohistochemistry

Paraffin sections of the small intestine were prepared after fixation with 4% paraformaldehyde for 12 h. The sections were placed in an 80°C thermostat and heated overnight. After that, xylene and alcohol was used for gradient dewaxing, 3% H_2_O_2_ was used to inactivate endogenous peroxidase, and ethylenediamine tetraacetic acid (EDTA) was used for antigen retrieval. Then, the sections were sealed with bovine serum albumin (BSA) to block nonspecific binding, and primary antibody was added and incubated with the sections at 4°C overnight. The primary antibodies used were anti-PCNA antibody (1:400, 13110, Cell Signaling Technology, Boston, United States), anti-Bmi1 antibody (1:200, ab126783, Abcam, Cambridge, United Kingdom), and anti-SOX9 antibody (1:1000, ab185966, Abcam, Cambridge, United Kingdom). On the second day, the sections were incubated with goat anti-rabbit secondary antibody (8114, Cell Signaling Technology, Boston, United States) at 37°C for 1 h without light. The sections were washed again with PBS, and the chromogenic substrate diaminobenzidine (DAB) (Zhongshan Golden Bridge Biotechnology, Beijing, China) was dropped on the sections. Staining was terminated when the positive cells were deeply stained but the background was unstained or lightly stained, as determined by viewing under a microscope. The sections were redyed with haematoxylin, fully rinsed with water, dehydrated with gradient alcohol and xylene, then sealed with the appropriate resin, dried, and photographed under an optical microscope (Nikon Corp. Tokyo, Japan). Semiquantitative analysis was performed with Image-Pro Plus 6.0 software.

### 2.9 Western blotting

Intestinal samples were thoroughly ground in liquid nitrogen and cleaved on ice for 5 min with an ultrasonic cell crusher (VCX130, Sonics, Connecticut, United States). The cleaved tissue was placed in a centrifuge tube and centrifuged with a rotational speed of 12000 ×g at 4°C for 20 min. The concentration of protein in the supernatant after centrifugation was measured by the BCA method. The protein solution was heated and denatured at 100°C for 5 min. The solution was removed, quickly placed on ice, and then stored at −20°C. Protein samples were transferred to a polyvinylidene fluoride (PVDF) membrane. Then, the PVDF membrane was sealed with milk for 90 min. The PVDF membrane was cleaned twice with TBST for 5 min each and incubated with primary antibodies at 4°C overnight. The primary antibodies used were as follows: anti-ANG1 antibody (1:500, A7877, ABclonal, Wuhan, China), anti-VEGF antibody (1:500, A12303, ABclonal, Wuhan, China), anti-IGF1 antibody (1:500, A11985, ABclonal, Wuhan, China), anti-Bcl-2 antibody (1:500, T40056, Abmart, Shanghai, China), anti-Bax antibody (1:1000, T40051, Abmart, Shanghai, China), and anti-β-actin antibody (1:1000, ab8226, Abcam, Cambridge, United Kingdom). On the second day, the membrane was incubated with horseradish peroxidase-conjugated secondary antibodies for 45 min at room temperature. Then, the PVDF membrane was soaked in enhanced chemiluminescence solution, and images were captured with a gel imaging system. Image analysis was performed with ImageJ software.

### 2.10 ELISA

The supernatant of the tissue lysis solution was obtained by the method used in Western blotting described above, and the concentrations of malondialdehyde (MDA), superoxide dismutase (SOD) and diamine oxidase (DAO) were detected with the following ELISA kits: MDA detection kit (CEA597Ge, Cloud-Clone Corp. Wuhan, China), SOD detection kit (SES134Ra, Cloud-Clone Corp. Wuhan, China), and DAO detection kit (SEA656Ra, Cloud-Clone Corp. Wuhan, China). All procedures were performed in accordance with the kit’s instructions, and the concentration of the index to be measured in the sample was finally calculated.

### 2.11 Statistical analysis

GraphPad Prism (8.3.0) statistical software was used for statistical analysis. The numerical data are expressed as the mean ± standard deviation (SD). Comparisons of differences between two groups were performed by *t*-test. One-way analysis of variance (ANOVA) was used for comparisons of differences among multiple groups. Differences for which *p* < 0.05 were considered statistically significant.

## 3 Results

### 3.1 Evaluation of the small intestinal IR injury model

**FIGURE 2:**
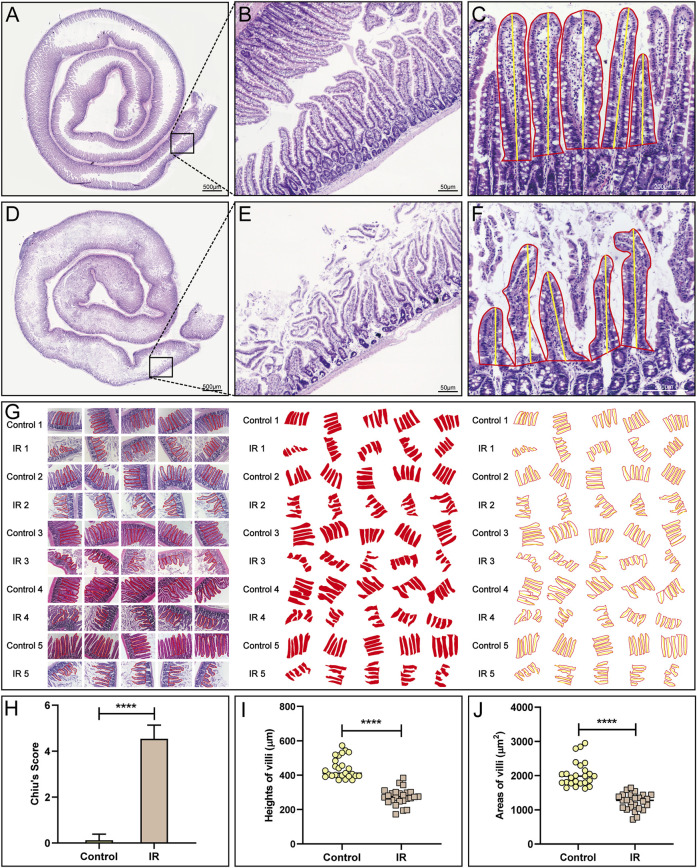
Pathological evaluation of the small intestinal IR injury model. **(A,B)** HE staining of the normal small intestine is shown. **(D,E)** HE staining of the modlled small intestine is shown. **(C,F,G)** The method **(C,F)** and process **(G)** used to measure villous height and area in the control and IR groups. **(H)** A statistical comparison of the small intestinal Chiu’s score between the control and IR groups was performed (*n* = 25). **(I,J)** A statistical comparison of the height and area of small intestinal villi between the control and IR groups was performed (*n* = 25). Data are presented as the mean ± SD (^****^ *p* < 0.0001).

The normal small intestine is red in color due to the adequate blood supply before the SMA was clamped (Figure 1C). After clamping of the SMA, the blood vessels supplying the intestinal tract narrowed, the intestinal tube immediately became pale, SMA pulsation gradually disappeared, and small intestinal peristalsis slowed (Figure 1D). After the clamp was opened, the SMA resumed pulsation, blood supply to the small intestine resumed, and the intestinal tube changed from pale to red in colour (Figure 1E). Comparison of small intestinal length between the IR group and the control group (Figure 1F) showed that in contrast to the control group, the IR group had a shorter small intestinal length (Figure 1G), and segments with ischemic necrosis were seen in the IR group. Pathological sections from the control and IR groups were prepared (Figures 2A,D), and the intestinal villi of the normal intestinal mucosa were found to be regular, continuous and dense (Figure 2B), while in the IR group, many small intestinal villi were damaged or ruptured, the lamina propria was destroyed, and the infiltration of a large number of inflammatory cells was observed (Figure 2E). The height and area of the small intestinal villi in the control group and IR group were measured under a microscope. The area of the small intestinal villi was marked in red, and the distance between the midpoint of the base of the small intestinal villi and the farthest point from the midpoint of the base was marked in yellow (Figures 2C,F). Figure 2G shows the process by which villous height and area were measured in the two groups. Statistical analysis showed that the Chiu’s score for the small intestine was significantly higher in the IR group relative to the control group (Figure 2H), indicating that this modelling method had damaged the intestinal mucosa. In the IR group, the villous height was lower, and the area was smaller than those of the control group (Figures 2I,J). Because the height and area of villi in the small intestine partly reflect intestinal mucosal function, this modelling method also destroyed intestinal function.

### 3.2 HFMSC isolation, culture and characterization

**FIGURE 3:**
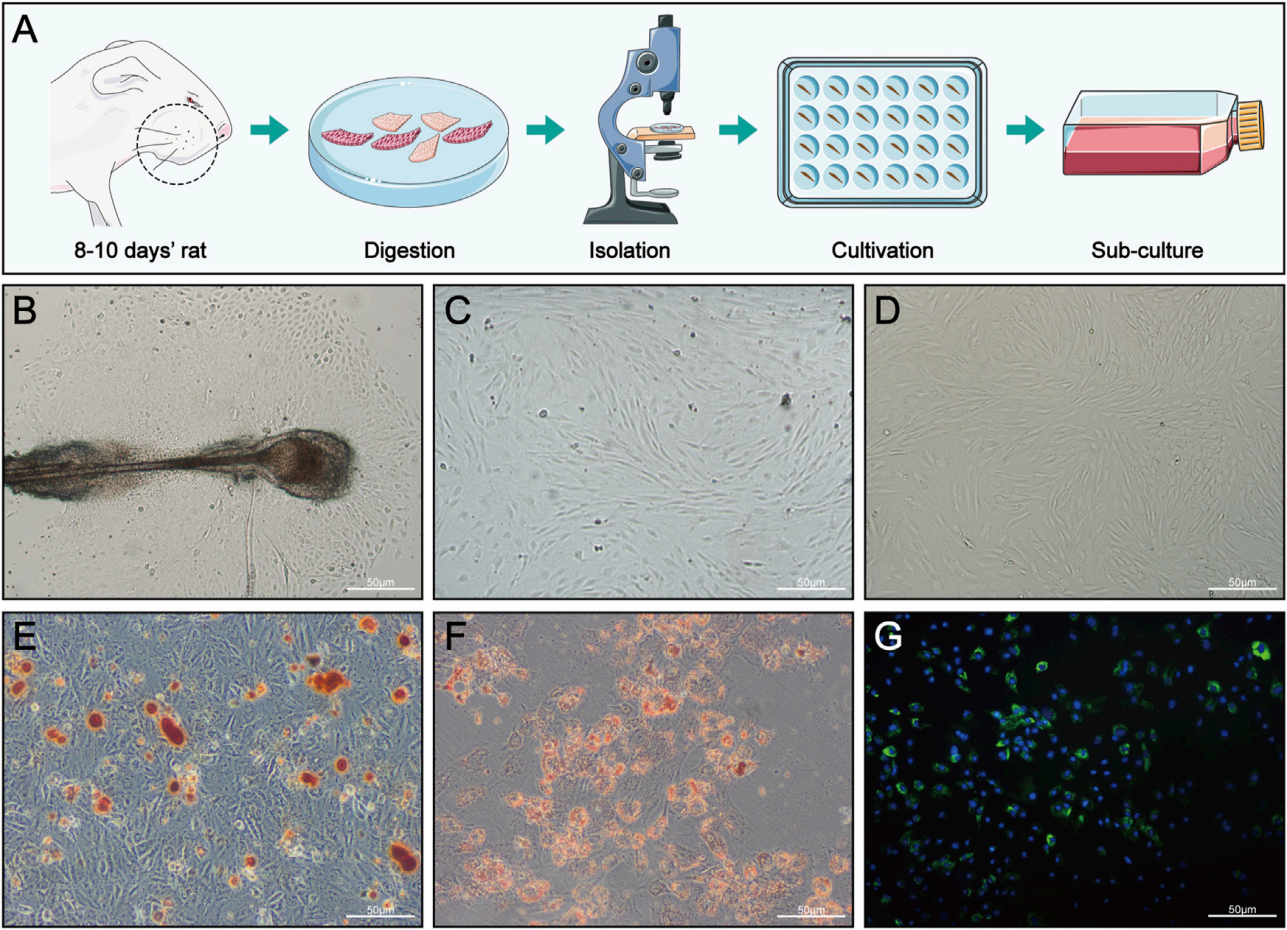
HFMSC isolation, culture, differentiation and staining. **(A)** Isolation and culture of HFMSCs. **(B)** Primary HFMSCs clustered around the dermal sheath and dermal papilla. **(C)** Primary cells cultured for approximately 10 days **(D)** First-passage of HFMSCs showed the classic fibroblast-like and adherent growth patterns of MSCs. **(E,F)** Osteogenic **(E)** and adipogenic **(F)** differentiation of HFMSCs. **(G)** The nuclei of HFMSCs were stained blue by DAPI, and the cell membrane was stained green by PKH67.

As shown in Figure 3B, the HFMSCs were dense and surrounded the dermal sheath and papilla. Primary cells cultured for approximately 10 days (Figure 3C) and the first-passage of cells (Figure 3D) showed the classic fibroblast-like and adherent growth patterns typical of MSCs. Assessments of osteogenic and adipogenic differentiation verified the multiple differentiation potential of HFMSCs. Mineralized nodules can be seen in Figure 3E, and fat droplets can be seen in Figure 3F. The nuclei of the HFMSCs were stained with DAPI, and the cell membrane was stained with PKH67 (Figure 3G). The above results indicated that HFMSCs were successfully extracted.

### 3.3 HFMSC potential to homing, proliferation and differentiation

**FIGURE 4:**
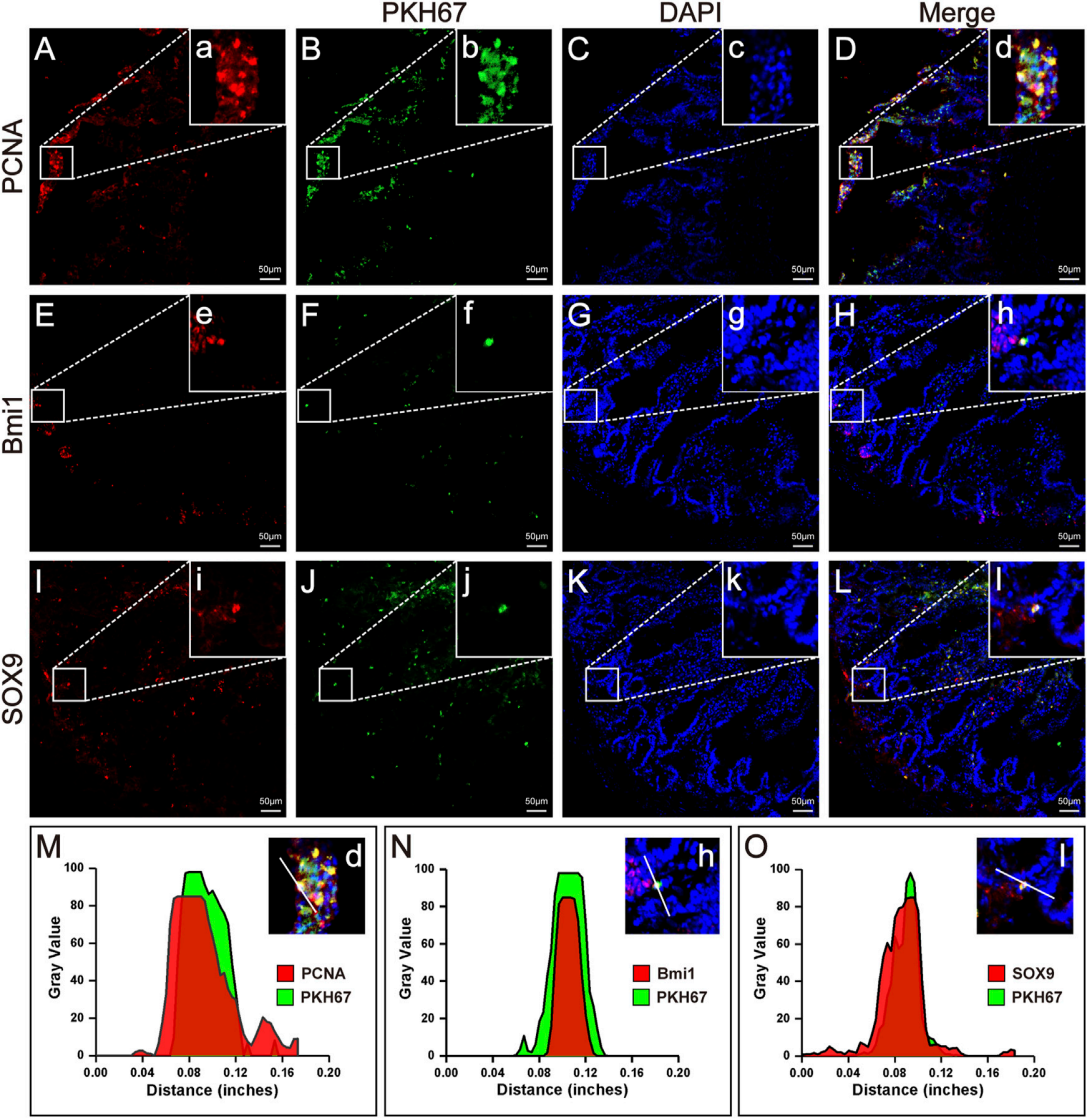
Homing, proliferation and differentiation of HFMSCs in IR + HFMSCs group. **(A,E,I)** Proliferating cell nuclear antigen PCNA **(A)** and the ISC markers Bmi1 **(E)** and SOX9 **(I)** emitted red fluorescence in small intestinal tissue. **(B, F, J)** PKH67-labeled HFMSCs homed to the damaged tissue site and emitted green fluorescence in small intestinal tissue. **(C,G,K)** DAPI-labeled cells emitted blue fluorescence. **(D,H,L)** Merging image from the red, green and blue fluorescent images. a-l Magnified image of the sites where PCNA, SO×9, Bmi1 and HFMSCs colocalized in A-L. **(M–O)** Cross-section fluorescence intensity analysis of the colocalization sites in **(D,H,L)**.

Immunofluorescence analysis under a fluorescence microscope showed the presence of HFMSCs labelled with PKH67 in the small intestinal tissue, which indicated that HFMSCs migrated to (homed to and remained at) the damaged site to exert their therapeutic effects (Figures 4B,F,J). We analysed the expression of proliferating cell nuclear antigen (PCNA) and two ISC markers, B cell-specific moloney murine leukemia virus Integration site-1 (Bmi1) and SRY-Box transcription factor-9 (SOX9), in the intestine. The above three proteins were bound to a fluorescence indicator (Figures 4A,E,I). Additionally, the nuclei were stained with DAPI (Figures 4C,G,K). We examined the fluorescence colocalization signal (Figures 4D,H,L) and analysed the overlap in fluorescence from the above three proteins and HFMSCs (Figures 4M–O), which proved that HFMSCs could express PCNA, Bmi1 and SOX9. These results indicated that HFMSCs not only homed to the damaged site, but also could colonize and proliferate in the intestinal tissue potentially and expressed ISC markers, indicating their potential to differentiate into small intestinal cells.

**FIGURE 5:**
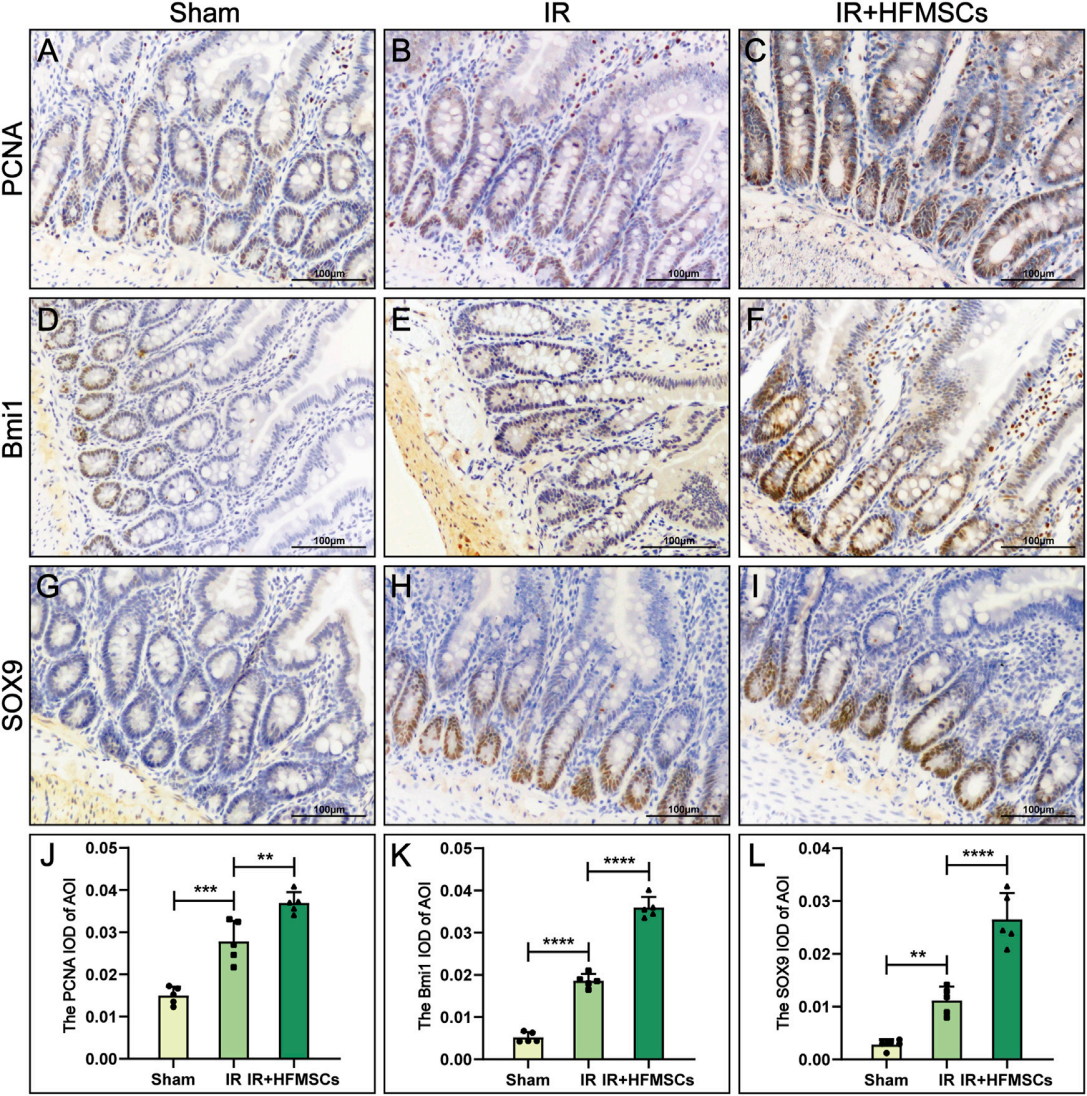
HFMSCs promoted the expression of PCNA, Bmi1 and SOX9 in the small intestine. **(A–I)** Expression of PCNA **(A–C)**, Bmi1 **(D–F)** and SOX9 **(H–J)** in the sham group (*n* = 5), IR group (*n* = 5) and IR + HFMSCs group (*n* = 5). **(J–L)** Statistical comparison of MODs of the AOIs of PCNA **(J)**, Bmi1 **(K)** and SOX9 **(L)** in the sham group, IR group and IR + HFMSCs group. The expression levels of PCNA, SOX9 and Bmi1 in the IR + HFMSCs group were the highest. Data are presented as the mean ± SD (^*^ *p* < 0.05, ^**^ *p* < 0.01, ^***^ *p* < 0.001, ^****^ *p* < 0.0001).

Semiquantitative immunohistochemical analysis of the small intestine revealed the expression of PCNA, Bmi1 and SOX9 (Figures 5A–I), and the mean optical densities (MODs) of these proteins in the area of interest (AOI) were measured. Based on the results, PCNA, Bmi1 and SOX9 were more expressed in the IR group relative to the sham group. The expression of the above three proteins in the IR + HFMSCs group was stronger than that in the IR group (Figure 5J–L).

### 3.4 HFMSCs potential to promote angiogenesis

**FIGURE 6:**
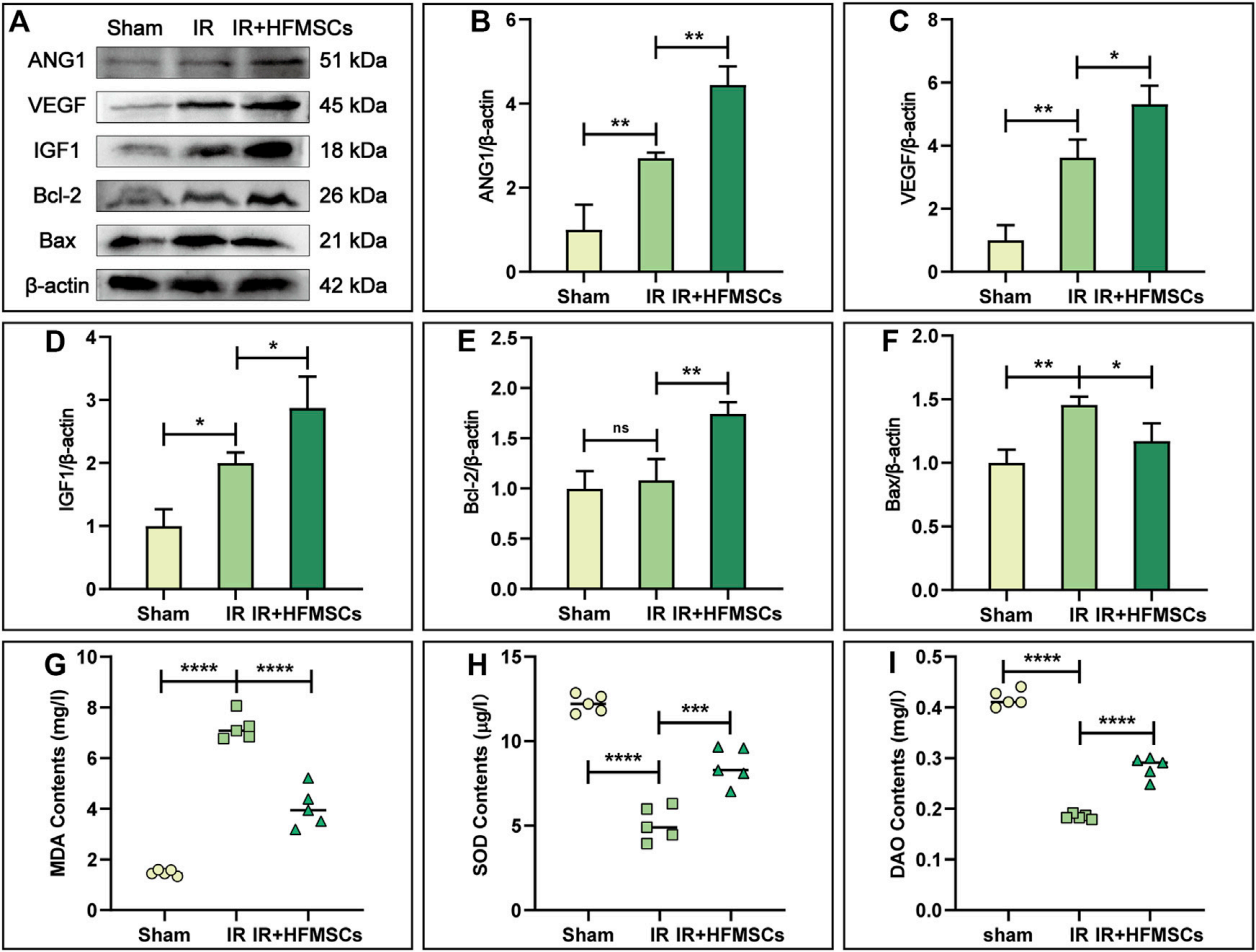
HFMSCs promoted angiogenesis, inhibited apoptosis, regulated oxidative stress and improved intestinal barrier function. **(A)** Representative Western blot images of ANG1, VEGF, IGF1, Bcl-2, and Bax in small intestinal tissues from different groups (*n* = 3). β-actin served as a reference. **(B–F)** Statistical analysis showed that ANG1 **(B)**, VEGF **(C)**, IGF1 **(D)** and Bcl-2 **(E)** expression levels were the highest in the IR + HFMSCs group, and Bax expression levels were lower in the IR + HFMSCs group than in the IR group **(F)**. **(G–I)** Statistical analysis of the MDA **(G)**, SOD **(H)** and DAO **(I)** levels in the small intestine in different groups (*n* = 5), as analysed by ELISA. The IR + HFMSCs group expressed less MDA than the IR group, whereas the IR + HFMSCs group expressed higher levels of SOD and DAO. Data are presented as the mean ± SD (^*^ *p* < 0.05, ^**^ *p* < 0.01, ^***^ *p* < 0.001, ^****^ *p* < 0.0001, ns indicates no significance).

Angiopoietin-1 (ANG1) and vascular endothelial growth factor (VEGF) are important substances that promote angiogenesis. A higher level of ANG1 and VEGF expression was observed in the IR group compared to the sham group (Figures 6A–C), indicating compensatory increases in ANG1 and VEGF levels after intestinal injury. The levels of ANG1 and VEGF were higher in the IR + HFMSCs group than in the IR group (Figures 6A–C). These results showed that HFMSCs were beneficial to the repair and regeneration of small intestinal vessels after IR.

### 3.5 HFMSCs potential to inhibit apoptosis

Insulin growth factor-1 (IGF1) is an important factor that facilitates the proliferation of intestinal epithelial cells and inhibits apoptosis. As shown by Western blotting, the level of IGF1 was highest in the IR + HFMSCs group, indicating that HFMSC treatment improved the expression of IGF1 in tissues (Figures 6A,D). B-cell lymphoma-2 (Bcl-2) and Bcl-2 homologous antagonist/killer (Bax) can reflect cell apoptosis. Although there was no significant difference in Bcl-2 expression between the sham and IR groups, Bax levels were higher in the IR group compared to the sham group. The expression level of Bcl-2 was higher in the IR + HFMSCs group relative to the IR group, whereas Bax expression level was lower in the IR + HFMSCs group (Figures 6A,E,F). These results indicated that the degree of intestinal cell apoptosis decreased significantly after HFMSC treatment.

### 3.6 HFMSCs potential to regulate oxidative stress and improve intestinal barrier function

The MDA and SOD content of the intestinal tissue were detected by ELISA. In the IR group, the MDA content was significantly higher than that in the sham group, while in the IR + HFMSCs group, the MDA content was low as compared to that in the IR group (Figure 6G). The SOD level in the IR group was lower than that in the sham group, and HFMSC treatment increased the SOD level (Figure 6H). In conclusion, HFMSCs were benefical to relieve reactive oxygen species (ROS) attack on the small intestine after IR. The level of the structural enzyme DAO in intestinal epithelial cells can reflect intestinal mucosal barrier function. The DAO content was significantly lower in the IR group than in the sham group, but it was higher in the IR + HFMSCs group than in the IR group (Figure 6I). Based on these results, HFMSC treatment can decrease IR damage to the intestinal mucosa potentially.

## 4 Discussion

The treatment of small intestinal IR injury is a challenging issue in clinical practice. The occurrence of ischemia is difficult to prevent, and the injury caused by reperfusion is inevitable. Along with the extensive application of MSCs in the regenerative medicine field, due to their stemness and safety, MSCs contribute to the treatment of many diseases, including IR and intestinal diseases (Hendijani, 2017). However, many stem cells from different sources have limitations. BMMSCs are difficult to obtain and require invasive methods (Abbaspanah et al., 2021). Adipose-derived MSCs (ADMSCs) are tightly bound to and not easy to separate from the extracellular matrix (Mazini et al., 2020). HFMSCs are easy to obtain, and the source is abundant. They can be obtained from donors without immune rejection without ethical problems (Wang et al., 2013). Moreover, HFMSCs have strong proliferative ability *in vitro* and can be cultured for 11–12 generations before cell ageing (Bajpai et al., 2012). These characteristics make HFMSCs much favoured among MSCs from different sources.

**FIGURE 7:**
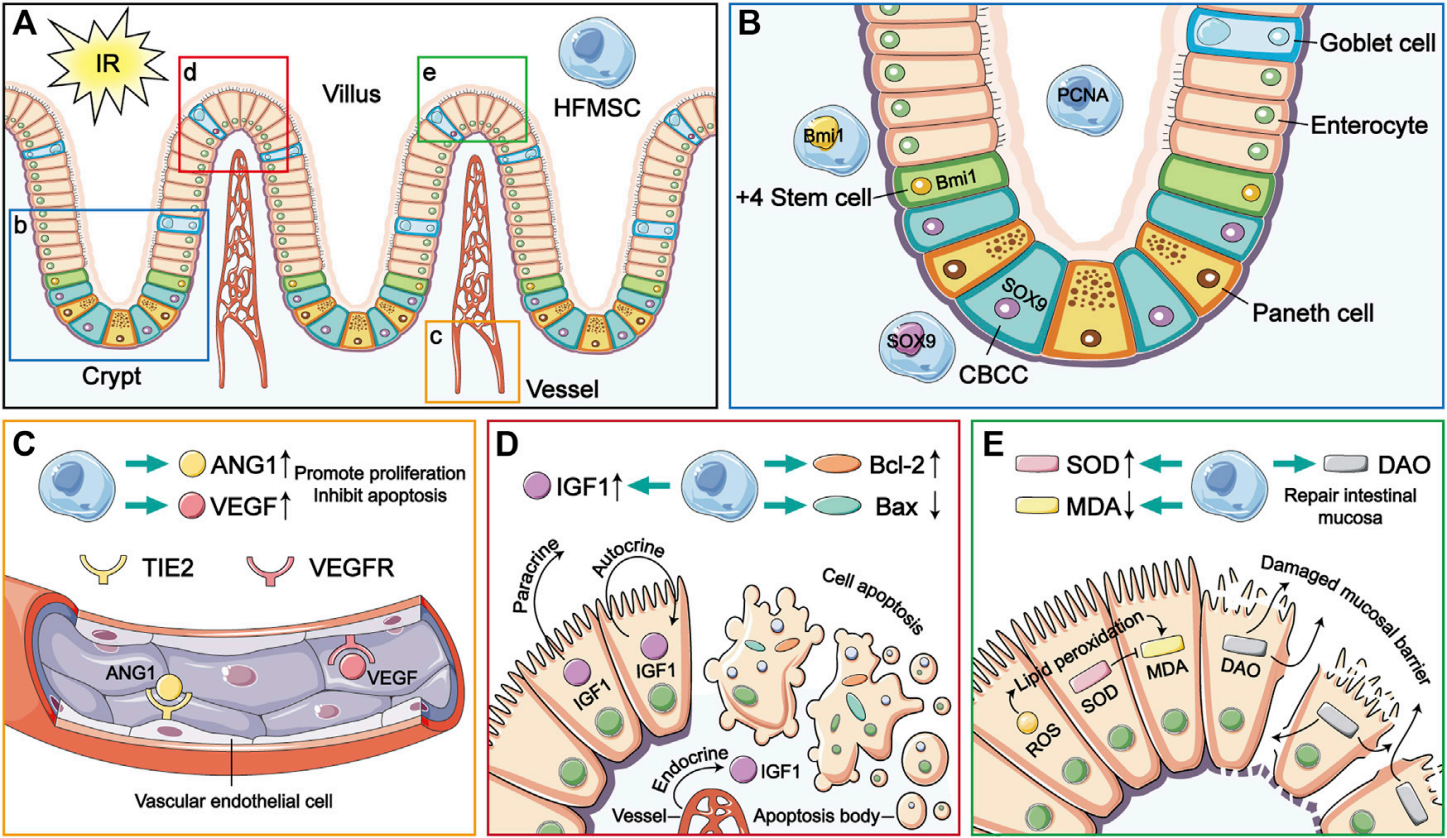
The therapeutic mechanism of HFMSCs in small intestinal IR injury. **(A)** HFMSCs home to the site damaged by IR in the small intestine. **(B–E)** are magnified versions of views b-e in **(A)**. **(B)** HFMSCs proliferated in the small intestine and expressed ISC markers. **(C)** HFMSCs increased the expression levels of ANG1 and VEGF in small intestinal tissues, promoting angiogenesis. TIE2 and VEGFR are receptors of ANG1 and VEGF, respectively. **(D)** HFMSCs increased the expression levels of IGF1 and Bcl-2 in the small intestine, reduced the expression levels of Bax, promoted proliferation and inhibited apoptosis. **(E)** HFMSCs reduced the MDA content and increased the SOD content, regulating oxidative stress levels in the IR damaged small intestine. HFMSCs increased the DAO content in the small intestine and protected mucosal barrier function.

Homing and multidirectional differentiation are recognized as intrinsic characteristics of MSCs (Liesveld et al., 2020; Tan et al., 2022). The homing function of MSCs involves their targeted migration to damaged organs for repair (Liesveld et al., 2020). Stem cells injected intravenously into experimental animals can cross the vascular endothelial barrier and reach the damaged site (Nitzsche et al., 2017). In this study, the results of immunofluorescence analysis showed that HFMSCs homed to intestinal tissue (Figure 7A) and expressed PCNA. PCNA is a nuclear proliferation antigen that participates in DNA replication and repair and can reflect the degree of cell proliferation (Cardano et al., 2020). The experimental results showed that transplanted HFMSCs had the potential to proliferate after colonization of into the intestine (Figure 7B).

As a kind of pluripotent stem cell, MSCs can be “reshaped” in different tissue environments and exhibit different phenotypes (Tan et al., 2022). After prior verification by our research team, the extracted HFMSCs were found to express MSC markers. Osteogenic and adipogenic differentiation was induced, which proved that HFMSCs retained multidirectional differentiation potential. Our results showed that HFMSCs could express ISC markers and participate in intestinal cell renewal. ISCs are the repository of intestinal cells used to satisfy the need for additional intestinal cells due to their fast renewal speed. Whenever the intestine is damaged, the ISCs in the crypts will fill upward (Nalapareddy et al., 2022). The position four cells from the bottom of the crypt is the source of ISCs, which are called +4 stem cells, and Bmi1 is a classic marker of these ISCs (Carulli et al., 2014). In addition, crypt base columnar cells (CBCCs) mixed with Paneth cells at the bottom of the crypt are also a very important kind of ISC, and SOX9 is not only a marker of CBCCs but also expressed by intestinal endocrine cells (Gracz et al., 2010; Van Landeghem et al., 2012). HFMSCs were found to express Bmi1 and SOX9, indicating that HFMSCs have the potential to differentiate into ISCs (Figure 7B), and after the HFMSCs were transplanted into the small intestine, the expression of ISC markers in the intestinal tissue increased, which can be conducive to intestinal cell turnover and mucosal repair.

The paracrine manner in which MSCs exert their various therapeutic effects has always been a research hotspot and is recognized as the main reason why MSCs are used to treat disease. MSCs can secrete a variety of cytokines to act on damaged tissues and organs (Chang et al., 2021). To comprehensively evaluate the therapeutic effect of the paracrine function of transplanted HFMSCs in a small intestinal IR model, we detected the levels of angiogenesis and assessed the ability of HFMSCs to inhibit apoptosis and regulate oxidative stress-related factors, which are involved in the mechanism of small intestinal IR injury.

In intestinal ischemia, leukocytes and platelets gather in vessels to block blood flow. A large number of inflammatory cytokines are released, and vascular endothelial cells swell, resulting in vasodilation and contraction dysfunction (Mester et al., 2018). ANG1 can promote the survival of vascular endothelial cells, inhibit endothelial cell apoptosis, and maintain vascular stability (Saharinen et al., 2017). During IR, VEGF can facilitate the proliferation of vascular endothelial cells, and regulate endothelial dysfunction, and also has antioxidant effects (Korkmaz et al., 2020). Previous experiments have proven that MSCs can release the beneficial cytokines ANG1 and VEGF (Takakura et al., 2000; Markel et al., 2015). Consistent with this, our experiment showed that HFMSCs could increase the expression levels of intestinal ANG1 and VEGF (Figure 7C). Therefore, transplanted HFMSCs can likely achieve a therapeutic effect in small intestinal IR injury through the repair of vessels.

Apoptosis-mediated programmed cell death is an important element that promotes IR damage (Ikeda et al., 1998). IGF1 is a kind of multifunctional regulatory factor that can facilitate proliferation and inhibit apoptosis in intestinal diseases. IGF1 in the small intestine is mainly secreted by the liver and delivered through blood in the form of endocrine. Small intestinal epithelial cells themselves can also synthesize IGF1 that works in a paracrine and autocrine manner. IGF1 can also facilitate angiogenesis and the regeneration of intestinal crypts, epithelial cells and ISCs (Bortvedt and Lund, 2012). Our results showed that HFMSCs could increase the level of IGF1 in the small intestine, which was consistent with previous experiments proving that MSCs can produce IGF1 (Chen et al., 2021). Bcl-2 is one of the key proteins that inhibit apoptosis. Bax, which belongs to the Bcl-2 family of proteins, can promote apoptosis (Ben-Shahar et al., 2021). Combined analysis of the levels of Bcl-2 and Bax in the different groups showed that the treatment of transplanted HFMSCs reduced intestinal cell apoptosis (Figure 7D).

The therapeutic effect of MSCs in oxidative stress injury is mostly attributed to a paracrine mechanism. MSCs not only have strong adaptability to oxidative stress, but can also produce antioxidants themselves and promote the production of antioxidants in IR-damaged tissues. MSCs have also been suggested to alleviate oxidative stress by donating their mitochondria to damaged tissues (Ikeda et al., 1998). For a long time, oxidative stress injury was thought to mainly be due to the production of a large amount of reactive oxygen species (ROS) (Gonzalez et al., 2015), which can activate the immune response and destroy the function of the intestinal barrier (Ben-Shahar et al., 2021). ROS attack the membrane and lead to lipid peroxidation. MDA is a degradation product of lipid peroxidation, and the MDA content reflects the level of oxidative stress injury (Tsikas, 2017). SOD is an important antioxidant enzyme that contributes to scavenging ROS (Kalogeris et al., 2016). HFMSCs reduced intestinal MDA levels, and the SOD content in the IR + HFMSCs group was also higher compared with the IR group, indicating that the transplanted HFMSCs is conducive to reduce the oxidative stress damage caused by IR.

In addition, ischemia leads to rupture of the connection between small intestinal epithelial cells and even the destruction of epithelial cells to separate them from the basement membrane. DAO, an important indicator of intestinal mucosal barrier function, is a structural enzyme found in intestinal epithelial cells. When intestinal mucosal epithelial cells are damaged, DAO is released from ruptured epithelial cells (Zhang et al., 2014; Meng et al., 2016). Therefore, the finding that treatment with transplanted HFMSCs reduced the release of DAO indicates its potential protective effect on intestinal mucosal barrier function (Figure 7E).

In this study, transplanted HFMSCs were applied for the treatment of small intestinal IR injury for the first time. The multifaceted roles of HFMSCs were verified as much as possible, and the various effects of HFMSCs in the context of IR were studied. However, the limitation of this study is that the molecular mechanism by which transplanted HFMSCs exert their therapeutic effect was not explored in-depth, and this issue needs to be further explored in the future.

## 5 Conclusion

This study proves the significance of transplanted HFMSCs in the treatment of small intestinal IR injury. In the context that HFMSCs have not been used in small intestinal IR injury in the past, the results show that transplanted HFMSCs home to the small intestine, proliferate, and differentiate into ISCs intrinsically, promote angiogenesis, reduce apoptosis, control the oxidative stress response via a paracrine mechanism and protect intestinal mucosal function potentially. In summary, our study provides a new choice for the treatment of small intestinal IR injury.

## Data Availability

The original contributions presented in the study are included in the article/Supplementary Material, further inquiries can be directed to the corresponding author.
